# Adverse childhood experiences, inflammation, and depression: evidence of sex- and stressor specific effects in a nationally representative longitudinal sample of U.S. adolescents

**DOI:** 10.1017/S0033291725001102

**Published:** 2025-05-13

**Authors:** Jay D. O’Shields, George M. Slavich, Orion Mowbray

**Affiliations:** 1 Department of Social Work, University of Alabama at Birmingham, Birmingham, AL, USA; 2 Department of Psychiatry and Biobehavioral Sciences, University of California, Los Angeles, CA, USA; 3 School of Social Work, University of Georgia, Athens, GA, USA

**Keywords:** adverse childhood experiences, C-reactive protein, depression, sex differences, stress

## Abstract

Although adverse childhood experiences (ACEs) are commonly associated with depressive symptoms in adulthood, studies frequently collapse ACEs into a single unitary index, making it difficult to identify specific targets for intervention and prevention. Furthermore, studies rarely explore sex differences in this area despite males and females often differing in the experiences of ACEs, depressive symptoms, and inflammatory activity. To address these issues, we used data from the National Longitudinal Study of Adolescent to Adult Health to model the effects of 10 different ACEs on C-reactive protein (CRP) and depressive symptoms in adulthood. Path modeling was used to measure the effects of ACEs on CRP and depressive symptoms conjointly while also assigning covariances among ACEs to assess their interrelations. Sex-by-ACE interaction terms and sex-disaggregated models were used to test for potential differences. Emotional abuse and parental incarceration were consistently related to both CRP and depressive symptoms for males and females. Childhood maltreatment was associated with depressive symptoms for females, whereas sexual abuse was associated with inflammation for males. Several covariances among ACEs were identified, indicating potential networks through which ACEs are indirectly associated with CRP and depressive symptoms. These data demonstrate that ACEs have differing direct effects on CRP and depressive symptoms – and that they differ with respect to how they cluster – for males versus females. These differences should be considered in theory and clinical workflows aiming to understand, treat, and prevent the long-term impacts of ACEs on depressive symptoms and inflammation-related health conditions in adulthood.

## Introduction

Adverse childhood experiences (ACEs) are a strong risk factor for the development of depressive symptoms in adulthood (Chapman et al., 2004; Tan & Mao, 2023). The first major ACEs study by Felitti et al. (1998) was notable in this context as it examined forms of adversity other than childhood abuse and neglect and found that long-term health outcomes are also strongly predicted by household dysfunction, such as witnessing violence, household substance use, familial mental health, and familial imprisonment. This study had a profound impact on stress research, as it also identified the types of ACEs most strongly associated with poor health and set the stage for using the ACEs questionnaire – and an ACE index – as a relatively simple way to quantify exposure to early-life adversity. Since then, evidence has accumulated showing that ACEs are associated with greater depressive symptoms starting in childhood and continuing into adulthood, and that the number of ACEs experienced is positively associated with the lifetime risk for developing depression (Chapman et al., 2004; Desch et al., 2023; Tan & Mao, 2023). Furthermore, 62% of adults who develop a major depressive episode (MDE) have experienced at least one ACE, and ACEs are also strongly associated with an increased lifetime risk for suicide (Dube et al., 2001; Thompson, Kingree, & Lamis, 2019).

One biological pathway through which ACEs may be related to depressive symptoms involves stress-related increases in inflammation (Slavich & Irwin, 2014). Experiences of early-life stressors calibrate the body’s neural threat detection and stress response systems (Slavich, 2020, 2022; Slavich et al., 2023b). In this context, ACEs can prime the body’s ‘fight-or-flight’ response, which can lead to immunosuppression and chronic inflammation if the stress response is activated frequently or prolonged (Slavich et al., 2023b). Increases in inflammatory signaling, in particular, are believed to influence neural activity in areas of the brain associated with mental health problems, including major depressive disorder (Dantzer et al., 2008). Repeated activation of the stress response can also induce epigenetic changes at the cellular level, increasing inflammatory activity associated with the stress response (Slavich & Cole, 2013; Slavich, Mengelkoch, & Cole, 2023a). Inflammatory activity and depressive symptoms are thought to be synergistic mechanisms, with inflammatory molecules affecting regions of the brain that initiate depressive symptoms, whereas negative mood states can lead to the activation of cognitive schemas that induce an inflammatory response (Messay, Lim, & Marsland, 2012; Beurel, Toups, & Nemeroff, 2020; Slavich et al., 2023a). Therefore, improving our understanding of how different ACEs conjointly affect inflammation and depression may provide new insights into how to develop interventions that most effectively reduce depression risk (Miller & Raison, 2016; Slavich, 2015).

### ACEs, inflammation & depression

Regrettably, understanding the associations between ACEs, inflammation, and depressive symptoms is difficult due to the frequent collapse of individual ACEs into a single, unitary index of total ACE exposure. Some of the earliest studies have justified this approach by emphasizing the interrelatedness of ACEs (Chapman et al., 2004). However, consistent with a stressor characteristics perspective on health (Slavich, 2016; Slavich et al., 2009; Slavich, O’Donovan, Epel, & Kemeny, 2010), recent evidence suggests that individual ACEs may have differential effects on biological and clinical outcomes (Alley, Gassen, & Slavich, 2025). For example, a history of family mental illness and sexual abuse may be some of the only significant ACEs associated with depressive symptoms in adulthood (Giano et al., 2021).

Meta-analytic results exploring associations between ACEs, inflammation, and depressive symptoms have only further underscored the need for a stressor characteristics perspective. For example, a meta-analysis of 22 studies showed that ACEs were positively associated with several inflammatory markers, including C-reactive protein (CRP), a protein produced by the liver that is frequently used as an index of chronic low-grade inflammation and correlates with cerebrospinal fluid samples of inflammatory cytokines implicated in depression (Felger et al. 2020; Orsolini et al. 2022; Zagaria et al. 2024). However, of the 13 studies included in this meta-analysis that included CRP, five used measures of childhood maltreatment only and did not include indicators of household dysfunction (such as incarceration of a parent) as commonly used in ACEs measurement (Chapman et al., 2004; Felitti et al., 1998). Therefore, the results of this meta-analysis may be more representative of the effects of childhood maltreatment, which has been identified as having a pro-inflammatory effect when explored outside an ACEs-specific framework (Kerr, McDonald, & Minnis, 2021). Furthermore, experiences of childhood maltreatment have been found to be associated with increased adipose tissue, proxied by BMI, which may be one pathway by which childhood maltreatment increases inflammation (see Moriarity, Mengelkoch, & Slavich, 2023); however, ACEs have been only weakly associated with obesity as indicated by a BMI of ≥30 (Hughes et al., 2017). Therefore, different ACEs may have different characteristics and, therefore, may relate to inflammation and depression differently.

### Sex differences

Further complicating the association between different ACEs, inflammation, and depressive symptoms is the multidimensional influence of sex. Whereas prior to puberty, males and females are approximately equally likely to experience depression, following the pubertal transition and up to menopause, females are roughly twice as likely to experience the disorder as compared to males (Nolen Hoeksema, 1987; Nolen-Hoeksema & Girgus, 1994; Slavich & Sacher, 2019). The fact that this doubling of depression risk occurs specifically following the pubertal transition and persists only during the reproductive years for females has led researchers to posit that female sex hormones that are released in greater quantities following puberty may affect inflammatory signaling in a manner that, in turn, increases depression risk in females (Lombardo, Mondelli, Dazzan, & Pariante, 2021; Slavich & Sacher, 2019). Additionally, females report greater rates of ACEs such as verbal abuse, physical abuse, and sexual abuse, which are associated with both increased inflammation and depressive symptoms (Derry et al., 2015; Keyes et al., 2012).

Despite these differences, sex differences in ACEs, inflammation, and depressive symptoms have not been widely investigated except for three prior studies. First, in a study of 85 U.S. college students, Kim, Watt, Ceballos, and Sharma (2019) found that collapsing ACEs into measures of abuse, neglect, and family dysfunction revealed a positive association between family dysfunction and CRP that was stronger for female students. Second, in a longitudinal study following English participants from 9 to 23 years old, Iob, Lacey, Giunchiglia, and Steptoe (2022) found that sexual abuse was associated with increases in CRP levels over time for boys but not girls. Finally, in a cross-sectional analysis of data from the Midlife in the United States study, Alley et al. (2025) found that childhood sexual abuse had a greater effect on CRP for females while having a similar overall increase in risk for depression in adulthood for both males and females.

### Study hypotheses

Based on the evidence reviewed above, we tested two key hypotheses: (a) individual ACEs would differentially predict inflammation and depressive symptoms, and (b) the relations predicted between ACEs, inflammation, and depressive symptoms would differ for males versus females. Given that longitudinal studies have identified inconsistent directionality in associations between ACEs clusters and trajectories of depressive symptoms over time, we further hypothesized that physical and emotional neglect would be associated with less inflammation and depressive symptoms as compared to the other ACEs (see Desch et al., 2023).

## Method

### Sample

The present study used data from the National Study of Adolescent to Adult Health (Add Health), a nationally representative panel study of the U.S. (Harris et al., 2019). The Add Health study began in 1994, sampling 90,118 adolescents in grades 7–12. From this pool, 20,745 participants were administered an in-home survey, forming the primary sampling frame for follow-up home surveys. Our analyses used data specifically from Waves 1 (1994–1995), 3 (2001–2002), and 4 (2008–2009), as well as the survey of parents at Wave 1 and the biomarker collection portion of Wave 4. The sample primarily identified as White (78.16%), non-Hispanic (9.27%), and female (64.63%). The mean age across each wave was 15.92 years (range: 12–20) at Wave 1, 21.78 years at Wave 3 (range: 18–26), and 28.92 years at Wave 4 (range: 24–32). No data came from Wave 2 due to new participants being surveyed during this wave that were not surveyed in Wave 1.

### Measures

***Adverse Childhood Experiences*** were measured similarly to prior studies that have explored the effects of ACEs in the Add Health data (Brumley, Jaffee, & Brumley, 2017; Schwartz, Wright, & Valgardson, 2019; Testa & Jackson, 2020). Specifically, the presence versus absence of ten different adversity categories were included: emotional abuse, physical abuse, sexual abuse, physical neglect, emotional neglect, parental incarceration, parental separation, substance use in the home, community violence, and exposure to suicide. The occurrence of each individual ACE was accounted for categorically as occurring (1) or not occurring (0). Emotional abuse, sexual abuse, physical abuse, and parental incarceration were assessed via participant self-report at Wave 4. Physical neglect was assessed via participant self-report at Wave 3. Community violence, emotional neglect, and exposure to family or friend suicide were assessed via participant self-report at Wave 1. Parental marital status was assessed via parent self-report at Wave 1. In-home substance use was assessed via a combination of participant and parent self-report at Wave 1. ACEs measures are described in Supplementary Table 1.

***Depressive symptoms*** were measured at Waves 1 and 4 using two versions of the modified Center for Epidemiological Studies-Depression scale (CES-D). At Wave 1, the modified CES-D was composed of 19 items, whereas Wave 4 was composed of 10 items. To maintain consistency in the measurement of depressive symptoms across waves, we only measured depressive symptoms using the 10 items measured consistently across Waves 1 and 4, which covered affective, cognitive, somatic, and interpersonal aspects of depressive symptoms. Each item asked the respondent to rate their experience of the symptom as 0 ‘rarely or none of the time (less than 1 day)’, 1 ‘some or a little of the time (1–2 days)’, 2 ‘occasionally or a moderate amount of the time (3–4 days)’, or 3 ‘most or all of the time (5–7 days)’. Items were summed to create a total score, with a potential range of 0–30, with higher scores indicating more depressive symptoms over the past week. Cronbach’s alpha was calculated based on the unweighted sample and was 0.85, indicating good internal consistency. A complete list of depressive symptom items can be reviewed in Supplementary Table 2.

***C-reactive protein*** levels at Wave 4 were used as a measure of inflammation. CRP levels were derived from capillary blood spot samples obtained via finger prick during an in-home visit. A single 3.2 mm diameter punch was taken from each obtained blood spot and placed in a deep-well microliter plate. Samples were then assayed via the sandwich enzyme-linked immunosorbent assay method (McDade et al., 2004). Sensitivity for assayed CRP was 0.035 mg/L. Within-assay coefficient of variation was 8.1%. Between-assay coefficient variation was 11.0% (Whitsel et al., 2012).

***Sex*** was assessed by participant self-report at Wave 1. Participants reported their sex as either female (1) or male (0). Because responses were constrained to a binary, rather than a continuous format that can account for gender as a spectrum of culturally masculine and feminine traits, we explicitly use the term ‘sex’ throughout this analysis.

***Control and demographic variables*** included several factors known to affect the association between CRP and depressive symptoms, as well as age, race, ethnicity, and socioeconomic indicators (Belsky et al., 2018; Belsky et al., 2019; Horn et al., 2018). Specifically, the control variables included BMI at Wave 4, presence of a chronic health condition (i.e., diabetes, heart disease, high blood cholesterol, high triglycerides, and high lipids) at Wave 4, any antidepressant use at Wave 4, any anti-inflammatory use at Wave 4, and exogenous sex hormone use at Wave 4. In turn, the demographic factors included self-reported racial identity at Wave 1 (White, Black, Asian and/or Pacific Islander, Native American, other racial identity, or multi-racial identity), self-reported ethnic identity at Wave 1 (Hispanic or non-Hispanic), and age at Wave 1. We include racial and ethnic identity for consistency with U.S. Census practices and to avoid unnecessary reduction of racial and ethnic data via the collapse of categories (Hirschman, Alba, & Farley, 2000; Ross, Hart-Johnson, Santen, & Zaidi, 2020).

Finally, we included four socioeconomic variables: Neighborhood socioeconomic disadvantage at Wave 1, Social Origins scores at Wave 1, Neighborhood socioeconomic disadvantages at Wave 4, and Occupational prestige scores at Wave 4. Social origins scores were a composite of parental education, parental occupation, household income, and household receipt of public assistance at Wave 1 (Belsky et al., 2018). Occupational prestige was an average of the Hauser and Warren Occupational Income and Occupational Education Scales at Wave 4 (Belsky et al., 2018). Neighborhood socioeconomic disadvantage was based on the proportion of five items: female-headed households, individuals living below the poverty threshold, individuals receiving public assistance, adults with less than a high school education, and adults who were unemployed within the participant’s census tract at Waves 1 and 4 (Belsky et al., 2019). A full description of these variables and their operationalization is in the Supplementary Materials, and a wave-by-wave summary of when each variable was collected is in Supplementary Figure 1.

### Analytic strategy

After combining the relevant data for Waves 1, 3, and 4 there were 4,900 participants in the data frame, of which 272 participants were removed due to not having a relevant survey weight. From the remaining 4,628 participants, 27 were removed due to parents of the participants not participating in the parent portion of the Wave 1 survey, leaving a final analytic sample of 4,601 participants. To control for the effects of acute infection, participants with CRP levels greater than 10 mg/L and at least one self-reported acute infection symptom within 2 weeks prior to blood sample collection were removed from the sample (Moriarity et al., 2021). Infection symptoms included flu symptoms, fever, night sweats, diarrhea, nausea, vomiting, bloody stool, polyuria, or skin rash. Among the remaining 3,615 participants in the analytic sample, 3,003 (83.07%) provided complete data. The greatest source of missingness was CRP (*n* = 189, 5.22%). A total of eight participants had missing data for depressive symptoms at Wave 4 (0.22%), whereas none had missing data for the presence of symptoms of infection. *T*-tests and chi-square analyses were used to assess the potential biasing effects of CRP missingness on the multivariate model. No association was found between missing values for CRP and depressive symptoms at Wave 4, ACE, gender, race, or ethnic identity. Therefore, all missing data were treated as missing at random.

**Figure 1.**
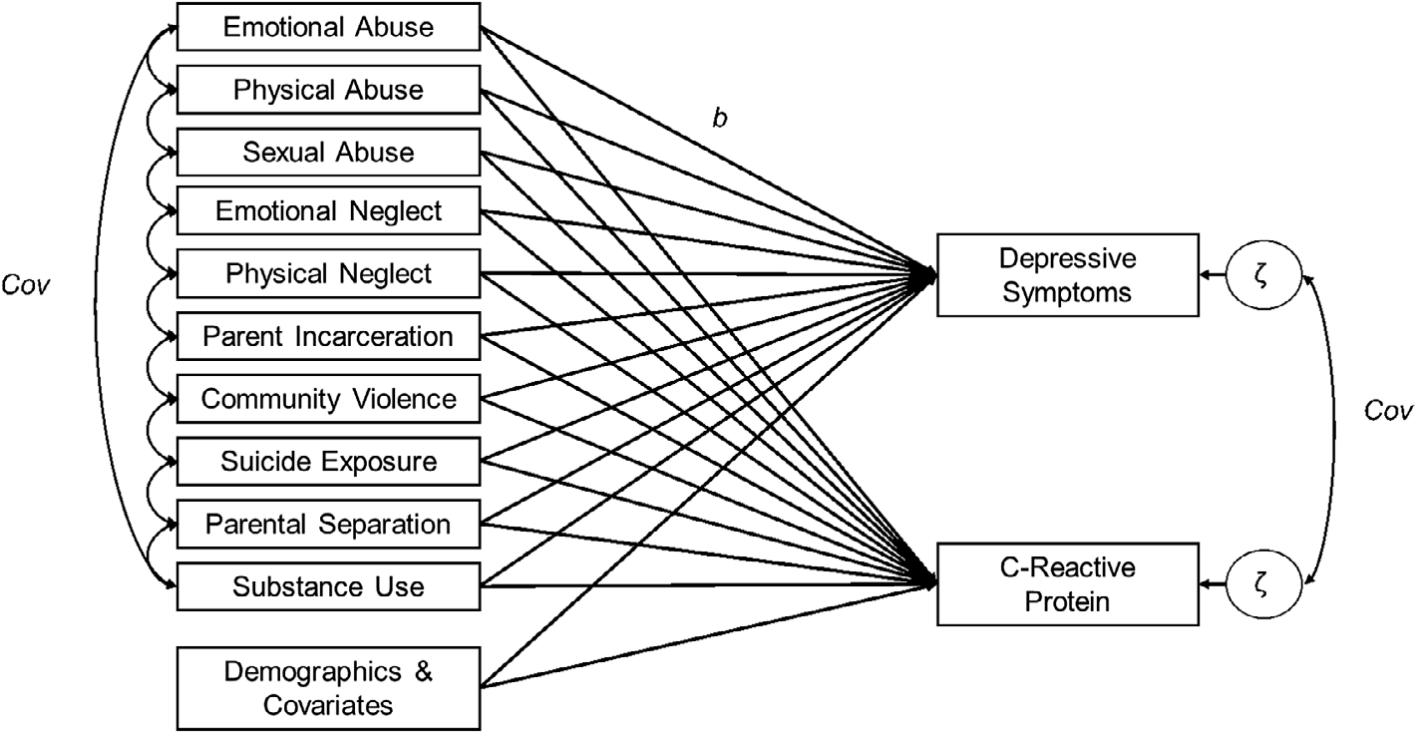
Depiction of the analytic model. Adverse childhood experiences (ACEs) are predictors C-reactive protein and depressive symptoms. Covariances between ACEs, as well as between the residuals of C-reactive protein and depressive symptoms, are also modeled. Some covariances between ACEs are omitted for visual clarity (e.g., emotional abuse ↔ sexual abuse, emotional abuse ↔ emotional neglect, physical abuse ↔ emotional neglect).

To investigate how individual ACEs were related to both depressive symptoms and CRP, we estimated three non-recursive path models across two phases of analyses. The first phase modeled each ACE as a predictor of depressive symptoms and CRP simultaneously in a multivariate model (that is all 10 ACEs in the same multivariate model). The association between CRP and depressive symptoms at Wave 4 was modeled via the covariance of the residuals of CRP and depressive symptoms. Depressive symptoms at Wave 1, self-reported sex, control variables, and demographic variables were included as covariates in the model, predicting depressive symptoms and CRP at Wave 4. BMI only predicted CRP, not depressive symptoms, per findings by Moriarity et al. (2023) that the inclusion of BMI as a covariate predicting depressive symptoms may increase the risk for false negative results when testing associations between inflammation and depression. To account for the interrelatedness of ACEs, covariances were assigned among each individual ACE. Additionally, because ACEs have been frequently associated with socioeconomic conditions, covariances were assigned between social origins score and neighborhood socioeconomic conditions at Wave 1 and individual ACEs. Lastly, the use of model fit indices was used to guide the addition of covariances between social origins score and neighborhood socioeconomic conditions at Wave 1, social origins score and occupational prestige score, and occupational prestige scores and neighborhood socioeconomic conditions at Wave 4. For a graphical representation of the analytic model, see Figure 1.

The second phase of analyses focused on identifying sex differences in the associations between ACEs, CRP, and depressive symptoms. We began by first disaggregating the sample by self-reported sex and then rerunning the non-recursive model as two separate models: male only and female only. Covariates for the sex-disaggregated models included control variables and demographic variables as previously implemented but did not include sex as a covariate. Covariances between ACEs and socioeconomic conditions were also retained for sex-disaggregated models. Notably, comparing sex-disaggregated models may identify differences in the magnitude of an association but not if the difference is statistically significant (Garcua-Sifuentes & Maney, 2021). Therefore, we re-pooled the males and females, testing for significance via the inclusion of a Sex × ACE interaction term for each ACE in a total sample multivariate model (Garcua-Sifuentes & Maney, 2021). Consistent with findings that not controlling for other interactions between sex and covariates could bias the results of the model (Keller, 2014), we also included interaction terms for all other variables in the model aside from exogenous sex hormone use and Hispanic ethnicity, as these resulted in a fully saturated model. Univariate sample statistics for the total sample as well as the sex-disaggregated samples were calculated to provide further context for the multivariate models, as were bivariate tests for the sex-disaggregated samples (*t*-tests and chi-square).

Analyses were conducted in R version 4.2.3 (R Core Team, 2021). Analyses accounted for the complex survey design elements of the Add Health dataset, as recommended by the Add Health team (Chen & Harris, 2020). This included accounting for the primary sampling unit (schools, *n* = 132), strata (census regions, *n* = 4), and sampling weight (Chen & Harris, 2020). To account for the longitudinal design of the analysis, we applied the sampling weight specified for longitudinal analysis of Waves 1, 3, and 4 (Chen & Harris, 2020). Univariate and bivariate analyses were conducted using the survey package recommended by Lumley (2024). Multivariate analyses were conducted using the Lavaan and lavaan.survey packages (Oberski, 2014; Rosseel, 2012).

Multivariate models were estimated using the robust maximum likelihood estimation method, accounting for the non-normal distribution of depressive symptoms and CRP in community samples. Due to the heightened risk for type II error inherent in subgroup comparisons (Garcua-Sifuentes & Maney, 2021), significance levels for Sex × ACE interaction terms were set at 0.0025 based on alpha at 0.05, correcting for 20 main-effect comparisons. To guide the interpretation of multivariate model fit, we report several model fit indices with cutoff scores based on recommendations by Kline (2005). These indices include model χ^2^ with a non-significant *p*-value indicating good fit, Comparative Fit Index (CFI) with acceptable fit being ≥0.90, Tucker-Lewis Index (TLI) with acceptable fit being ≥0.95, Standardized Root Mean Square Residual (SRMSR) with acceptable fit being <0.08, and Root Mean Square Error of Approximation (RMSEA) with acceptable fit being <0.08. Additionally, we report model χ^2^ divided by degrees of freedom, Akaike Information Criterion (AIC), Bayesian Information Criterion (BIC), and Loglikelihood values, which do not have a cutoff to indicate model fit but can be used to facilitate comparisons between the male and female models. Lastly, model fit indices can diverge as the number of observed variables increases, with CFI and TLI worsening while RMSEA improves with large numbers of observed variables (Shi, Lee, & Maydeu-Olivares, 2019). Therefore, we also provide the baseline model χ^2^(*df*) to serve as a comparator to the results obtained from our multivariate models.

## Results

### Descriptive statistics

Mean depressive symptoms for the sample were relatively low (*M* = 6.48, *SE* = 0.16). Mean CRP levels (*M* = 4.66, *SE* = 0.16) were above the threshold of 3.0 mg/L for low-grade inflammation with a right skew, although median CRP levels were 2.56. There was no significant difference in depressive symptoms between Wave 1 (*M* = 6.57, *SE* = 0.11) and Wave 4 (*M* = 6.48, *SE* = 0.11), *t*(127) = −0.85, *p* = 0.39. In terms of early adversity, the most common ACE experienced was emotional abuse (48.59%) followed by in-home substance use (17.15%), whereas the least common was suicide exposure (3.77%). Most of the sample experienced at least 1 ACE (69.86%), although 30.14% experienced no ACEs. These prevalence rates are similar to a recent Centers for Disease Control and Prevention report of U.S. adults, which found that 36.1% of the population experienced no ACEs, with the most common ACE being emotional abuse (34%) (see Swedo et al., 2023, Tables 2 and 3). A complete summary of the sample descriptive statistics can be found in Table 1, and comparisons for males versus females is presented in Table 2.

**Table 1.** Descriptive statistics

	Mean (*SE*) or %
Depressive symptoms	
Depressive symptoms at Wave 1	6.57 (0.11)
Depressive symptoms at Wave 4	6.48 (0.11)
Inflammation	
C-reactive protein at Wave 4	4.66 (0.16)
ACEs	
Emotional abuse	48.59%
Physical abuse	17.04%
Sexual abuse	5.33%
Physical neglect	9.75%
Emotional neglect	15.40%
Parental separation	16.99%
In-home substance use	17.15%
Parental incarceration	10.37%
Suicide exposure	3.77%
Community violence	9.39%
Control variable	
Depressive symptoms at Wave 1	10.82 (0.17)
Chronic health condition at Wave 4	18.30%
Antidepressant at Wave 4	15.18%
Anti-inflammatory at Wave 4	38.63%
Exogenous sex hormone at Wave 4	27.89%
Current tobacco cigarette smoking at Wave 4	33.28%
BMI at Wave 4	28.85 (0.22)
Demographics	
Age at Wave 1	15.92 (0.11)
Race: White	78.16%
Race: Black	10.28%
Race: Asian/Pacific Islander	1.79%
Race: Native American	0.49%
Race: Other racial identity	5.35%
Race: Multi racial identity	3.90%
Ethnicity: Hispanic	9.27%
Sex: female	64.63%
Social origins score	0.15 (0.07)
Occupational prestige score	99.51 (1.24)
Neighborhood at Wave 1	23.87 (0.85)
Neighborhood at Wave 4	20.01 (0.39)

**Table 2.** Mean and frequency comparisons for female versus male participants

	Female	Male	
	Mean (*SE*) or *N (%)*	Mean (*SE*) or *N (%)*	Test statistic (*df*)
*Depressive symptoms*			
Depressive symptoms (Wave 1)	6.92 (0.14)	5.16 (0.14)	4.80 (127)***
Depressive symptoms (Wave 4)	6.52 (0.13)	6.41 (0.20)	0.49 (127)
Inflammation			
C-reactive protein mg/L (Wave 4)	5.42 (0.21)	3.27 (0.20)	7.43 (127)***
*ACE*			
Emotional abuse	2,218,138 (33.43%)	1,005,428 (15.15%)	14.29 (128)***
Physical abuse	699,864.4 (10.54%)	430,586.8 (6.49%)	0.80 (128)
Sexual abuse	290,454.77 (4.37%)	63,325.35 (0.95%)	10.89 (128)**
Physical neglect	298,999.8 (4.50%)	326,808.3 (4.92%)	19.24 (128)***
Emotional neglect	764,319.9 (11.52%)	246,861.2 (3.72%)	14.35 (128)***
Parental separation	734,983.9 (11.07%)	392,617.5 (5.91%)	0.06 (128)
In-home substance use	718,088.0 (10.82%)	405,546.4 (6.11%)	0.06 (128)
Parental incarceration	423,638.6 (6.38%)	264,271.6 (3.98%)	1.01 (128)
Suicide exposure	179,073.74 (2.69%)	71,452.74 (1.07%)	1.94 (128)
Community violence	303,416.8 (4.57%)	314,669.2 (4.74%)	14.84 (128)***
*Control variables*			
Chronic health condition (Wave 4)	681,947.1 (10.27%)	532,289.8 (8.02%)	9.61 (128)**
Antidepressant (Wave 4)	662,274.1 (9.98%)	344,824.2 (5.19%)	0.19 (128)
Anti-inflammatory (Wave 4)	1,651,193.8 (24.89%)	912,081.8 (13.74%)	0.02 (128)
Exogenous sex hormone (Wave 4)	1,943,090.807 (29.29%)	7,614.795 (0.11%)	258.07 (128)***
Current tobacco cigarette smoking (Wave 4)	1,365,387.7 (20.58%)	842,338.6 (12.69%)	34.92 (128)***
Body mass index (Wave 4)	28.46 (0.28)	29.58 (0.31)	−2.84 (127)**
*Demographics*			
Age (Wave 1)	15.79 (0.12)	16.15 (0.13)	−3.42 (127)***
Race: White	3,355,559.1 (50.58%)	1,824,542.5 (27.50%)	1.08 (124)
Race: Black	457,546.9 (6.89%)	223,811.2 (3.37%)	
Race: Asian/Pacific Islander	87,395.06 (1.31%)	31,345.67 (0.47%)	
Race: Native American	19,162.24 (0.28%)	13,872.68 (0.20%)	
Race: Other racial identity	214,500.4 (3.23%)	140,514.8 (2.11%)	
Race: Multi racial identity	146,534 (2.20%)	112,225.3 (1.69%)	
Ethnicity: Hispanic	355,852.7 (5.36%)	259,440.3 (3.91%)	3.97 (128)*
Ethnicity: non-Hispanic	3,931,676.7 (59.26%)	2,086,853.8 (3.14%)	
Social origins score (Wave 1)	0.18 (0.07)	0.09 (0.09)	1.13 (127)
Occupational prestige score (Wave 4)	102.66 (1.22)	93.75 (2.10)	4.48 (126)***
Neighborhood (Wave 1)	23.80 (0.86)	23.99 (1.02)	−0.27 (127)
Neighborhood (Wave 4)	19.72 (0.40)	20.56 (0.50)	−2.06 (127)*

*Note:* Analyses accounted for complex survey design elements and therefore degrees of freedom are based on the number of primary sampling units and strata (that is design based degrees of freedom). All values are based on the weighted sample.

*p < 0.05, **p < 0.01, ***p < 0.001

### Multivariate analysis

The RMSEA (0.05) and SRMR (0.05) both showed excellent fit. However, model χ^2^ (2369.88, *df* = 233), CFI (0.67), and TLI (0.49) each indicated a poor fit. As hypothesized, several ACEs differentially predicted depressive symptoms and CRP. Emotional abuse was related to greater depressive symptoms (*b* = 0.82, SE = 0.17, *p* < 0.001) but lower CRP levels (*b* = −0.79, *SE* = 0.24, *p* < 0.001). Sexual abuse was related to greater depressive symptoms (*b* = 1.02, *SE* = 0.34, *p* < 0.01) but was not associated with CRP (*p* = 0.78). Parental incarceration was associated with higher CRP levels (*b* = 1.96, *SE* = 0.40, *p* < 0.001) but unrelated to depressive symptoms (*p* = 0.76). Exposure to suicide was associated with greater depressive symptoms (*b* = 1.22, *SE* = 0.39, *p* < 0.01) but unrelated to CRP (*p* = 0.44). Finally, CRP levels were not significantly related to depressive symptoms (*p* = 0.84). A complete report of fit indices can be found in Table 3, and associations between individual ACEs, depressive symptoms, and CRP are presented in Table 4, with standardized betas presented in Supplementary Table 3.

**Table 3.** Comparative model fit indices for multivariate models

	Total Sample (*N* = 6,633,824)	Females (*N* = 4,287,530)	Males (*N* = 2,346,294)
Baseline χ^2^(*df*)	6898.70 (360)***	5004.237 (344)***	2454.13 (344)***
χ^2^(*df*)	2369.81 (233)***	1842.77 (219)***	809.31 (219)***
χ^2^/*df*	10.17	8.41	3.69
CFI	0.67	0.65	0.72
TLI	0.49	0.45	0.56
RMSEA	0.05	0.05	0.05
SRMR	0.05	0.05	0.05
AIC	134,288.28	94,570.62	38,482.13
BIC	135,243.50	95,458.81	39,234.007
Loglikelihood	−66,985.14	−47,128.31	−19,084.06

*Note;* Analyses accounted for complex survey design elements and therefore degrees of freedom are based on the number of primary sampling units and strata (that is design based degrees of freedom). All values are based on the weighted sample.

Abbreviations: CFI, comparative fit index; TLI, Tucker-Lewis index; RMSEA, root mean square error of approximation; SRMR, standardized root mean square residual; AIC, Akaike information criterion; BIC, Bayesian information criterion

***p < 0.001

**Table 4.** Coefficients for non-recursive path model of different ACEs predicting depressive symptoms and CRP across the total sample, females only, and males only

	Total		Females		Males	
	Depressive symptoms	CRP	Depressive symptoms	CRP	Depressive symptoms	CRP
	*b* (*SE*)	*b* (*SE*)	*b* (*SE*)	*b* (*SE*)	*b* (*SE*)	*b* (*SE*)
ACE						
Emotional abuse	0.82 (0.17)***	−0.79 (0.24)***	0.73 (0.20)***	−0.96 (0.34)**	0.87 (0.29)**	−0.63 (0.24)**
Physical abuse	0.26 (0.22)	−0.25 (0.32)	0.72 (0.27)**	−0.04 (0.46)	−0.57 (0.36)	−0.14 (0.31)
Sexual abuse	1.024 (0.34)**	−0.13 (0.50)	1.18 (0.37)**	−0.60 (0.62)	0.03 (0.82)	2.67 (0.69)***
Physical neglect	−0.56 (0.27)	−0.36 (0.39)	−0.74 (0.38)	−1.03 (0.63)	−0.52 (0.39)	0.60 (0.33)
Emotional neglect	0.56 (0.22)	−0.04 (0.33)	0.77 (0.26)**	0.08 (0.43)	−0.14 (0.43)	−0.44 (0.36)
Parental separation	−0.01 (0.21)	−0.42 (0.31)	0.06 (0.26)	−0.59 (0.43)	−0.20 (0.38)	0.02 (0.32)
In-home substance use	0.15 (0.22)	0.54 (0.32)	0.21 (0.26)	0.65 (0.43)	0.21 (0.40)	0.45 (0.34)
Parental incarceration	−0.08 (0.27)	1.96 (0.40)***	−0.35 (0.32)	2.09 (0.55)***	0.69 (0.47)	0.90 (0.40)*
Suicide exposure	1.22 (0.39)**	0.44 (0.58)	−0.21 (0.46)	0.45 (0.77)	4.74 (0.73)***	−0.24 (0.61)
Community violence	−0.07 (0.27)	−0.14 (0.40)	−0.25 (0.38)	−0.18 (0.64)	−0.07 (0.41)	−0.01 (0.34)

*Note:* Analyses accounted for complex survey design elements and therefore standard errors are based on the number of primary sampling units and strata (that is design based degrees of freedom). All values are based on the weighted sample.

Abbreviations: ACE, adverse childhood experience; CRP, C-reactive protein.

**p < 0.01, ***p < 0.001

Several covariances between ACEs were significant, with exposure to suicide showing the weakest covariance associations among ACEs. Most notably, the only significant covariances with suicide exposure were emotional abuse (*Cov* = 0.07, *SE* = 0.002, *p* < 0.001), physical abuse (*Cov* = 0.004, *SE* = 0.001, *p* < 0.001), and emotional neglect (*Cov* = 0.003, *SE* = 0.001, *p* < 0.01). The strongest covariances among ACEs were found between emotional abuse and physical abuse (*Cov* = 0.06, *SE* = 0.004, *p* < 0.001), emotional abuse and emotional neglect (*Cov* = 0.03, *SE* = 0.003, *p* < 0.001), and in-home substance use and parental incarceration (*Cov* = 0.02, *SE* = 0.002, *p* < 0.001). A matrix of covariances between ACEs, as well as covariance for the residuals of CRP and depressive symptoms for the total model, is shown in Supplementary Table 4.

### Sex disaggregated models

Fit indices showed similar mixed findings across sex-disaggregated models. The RMSEA (female: 0.06; male: 0.05) and SRMR (female: 0.05; male: 0.05) both indicated excellent model fit. However, model χ^2^ (female: χ^2^ = 1873.92, *df* = 219; male: χ^2^ = 809.31, *df* = 219), CFI (female: 0.64; male: 0.72), and TLI (female: 0.44; male: 0.56) each indicated a poor fit. Consistent with our hypothesis, there were several differences between the male-only and female-only models in terms of associations between ACEs, depressive symptoms, and CRP. For both males and females, emotional abuse was associated with greater depressive symptoms (female: *b* = 0.72, *SE* = 0.20, *p* < 0.001; male: *b* = 0.87, *SE* = 0.29, *p* < 0.01) but lower CRP levels (female: *b* = −0.96, *SE* = 0.34, *p* < 0.01; male: *b* = −0.63, *SE* = 0.24, *p* < 0.05). Similarly, parental incarceration was related to higher CRP levels for both groups (female: *b* = 2.098, *SE* = 0.55, *p* < 0.001; male: *b* = 0.90, *SE* = 0.40, *p* < 0.05). Physical abuse and sexual abuse were both related to greater depressive symptoms, but only for females (*b* = 0.05, *SE* = 0.27, *p* < 0.01, and *b* = 0.06, *SE* = 0.37, *p* < 0.01, respectively); in turn, sexual abuse was associated with CRP levels, but only for males (*b* = 2.67, *SE* = 0.69, *p* < 0.001). For females, emotional neglect was related to greater depressive symptoms (*b* = 0.05, *SE* = 0.26, *p* < 0.01), but no relation was found for males (*p* = 0.73). Exposure to suicide was the strongest predictor across all models and was related to greater depressive symptoms, but only for males (*b* = 4.74, *SE* = 0.73, *p* < 0.001). CRP levels and depressive symptoms were not interrelated for females (*p* = 0.80) or males (*p* = 0.133).

**Figure 2.**
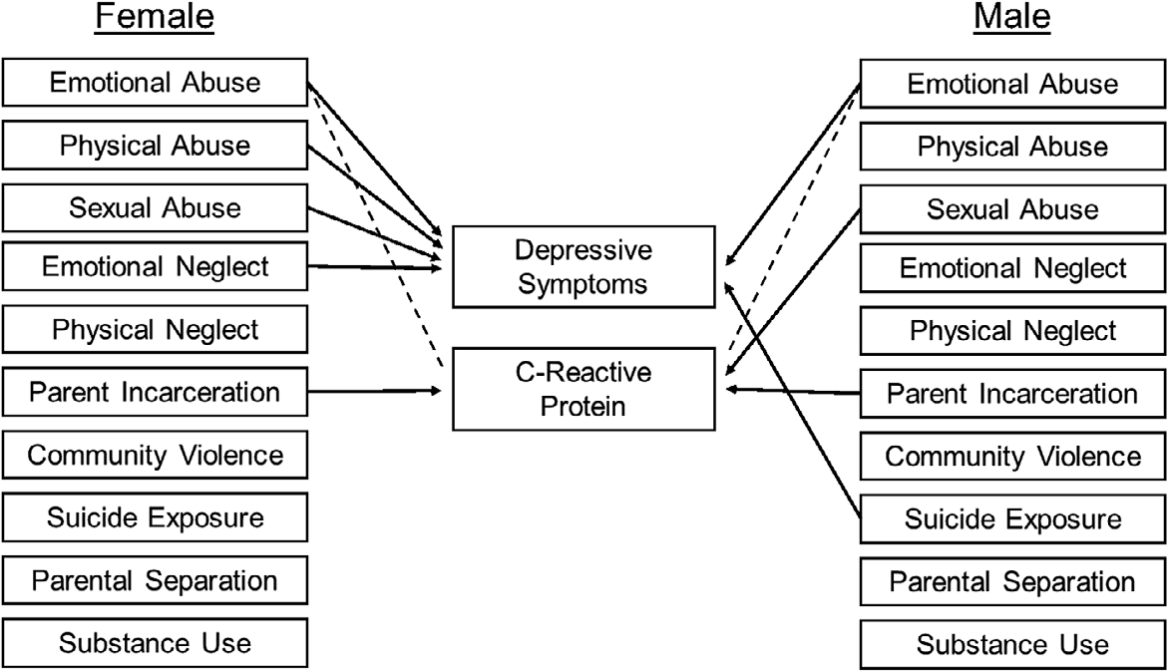
Associations between adverse childhood experiences (ACEs), C-reactive protein, and depressive symptoms. Only significant paths are presented. Positive associations are depicted by a solid line. Negative associations are depicted with a dashed line. Covariances are omitted for visual clarity. Betas and standard errors are presented in Table 4.

As with the total sample model, exposure to suicide exhibited the weakest covariance with the other ACEs in the female model, being only associated with community violence (*Cov* = 0.14, *SE* = 0.01, *p* < 0.001). Also similar to the total model, emotional abuse and physical abuse had the strongest covariance among ACEs for both males and females (female: *Cov* = 0.35, *SE* = 0.01, *p* < 0.001; male: *Cov* = 0.70, *SE* = 0.07, *p* < 0.001). However, for males, we found particularly strong covariance between in-home substance use and parental incarceration (*Cov* = 0.03, *SE* = 0.004, *p* < 0.001), and in-home substance use and parental separation (*Cov* = 0.04, *SE* = 0.005, *p* < 0.01). Covariances between all ACEs, as well as the residuals of depressive symptoms and CRP, can be found in Supplementary Table 4, and a graphical depiction of the sex-differences model is shown in Figure 2. Finally, a matrix of covariances is in Supplementary Tables 5 and 6.

### Significance testing for multivariate sex differences

Because disaggregating models by sex alone does not indicate if differences between the models are significantly different, we tested Sex × ACE type interaction terms in the combined multivariate model to identify if the effect of each ACE on CRP and depressive symptoms at Wave 4 differed by sex. Several significant interaction terms were identified, indicating significant sex differences. The effect of sexual abuse on CRP were significantly greater for males versus females (*b* = −3.38, *SE* = 0.57, *p* < 0.001). The effect of physical abuse on depressive symptoms was greater for females versus males (*b* = 1.27, *SE* = 0.27, *p* < 0.001), as was the effect of sexual abuse on depressive symptoms (*b* = 1.17, *SE* = 0.37, *p* < 0.0025). The effect of suicide exposure on depressive symptoms was greater for males versus females (*b* = −4.96, *SE* = 0.47, *p* < 0.001). While each of the above associations survived adjustment for multiple comparisons, the association between physical neglect and CRP did not (*b* = −1.60, *SE* = 0.57, *p* = 0.005). Therefore, significance testing of interaction terms confirms the results observed in the sex-disaggregated models.

## Discussion

The present study is the first that we know of to use nationally representative data of the United States to characterize longitudinal associations between individual ACEs, CRP levels, and depressive symptoms in adulthood. Consistent with a stressor characteristics perspective (Slavich, 2016; Slavich et al., 2009; 2010), we found that different ACEs are not uniformly related to CRP levels and depressive symptoms but, rather, differ substantially by ACE. Moreover, although we found some similar patterns for females and males, this was more the exception than the rule. Whereas emotional abuse and parental incarceration were similarly associated with depressive symptoms and CRP, for example, experiences of abuse, emotional neglect, and exposure to suicide were differentially related to these outcomes. Differences also emerged in how ACE exposures clustered together for males versus females, indicating that theory and research on ACEs should account for potential sex differences.

More broadly, although a large literature has emerged documenting a general association of ACEs with depression in adulthood, the frequent collapse of ACEs into a single unitary total ACE exposure score has made it impossible to understand how different ACEs may be differentially associated with depression. Furthermore, ACEs may be differentially related to physiological pathways such as inflammation, which may help us understand how ACEs ultimately lead to depression (Slavich & Auerbach, 2018; Slavich & Sacher, 2019). The present study adds to the small but growing number of studies showing differences in associations between ACEs, inflammation, and depressive symptoms for men and women (e.g., Iob et al., 2022; Kim et al., 2019; Lacey, Pinto Pereira, Li, & Danese, 2020).

Most notably, Iob et al. (2022) tested associations between individual ACEs, CRP, and depressive symptoms in a longitudinal study of English-identifying children, following them until age 23. Our data are consistent with Iob et al. (2022) who found that sexual abuse appears to be associated with a significant prospective increase in CRP, with the present data thus extending Iob et al.’s findings by replicating the association in males in the U.S. between the ages of 24–32. Our findings contrast with Lacey et al. (2020) who did not find an association between sexual abuse and CRP in a 1958 British birth cohort study, although that sample was 44–45 years old when CRP was assessed and no sex differences were investigated. Interestingly, our findings were also not replicated by Kim et al. (2019), who similarly assessed the effects of ACEs on depressive symptoms and CRP in a sample of 85 undergraduate students but who collapsed abuse into a single broad category. Results of the present study are thus generally consistent with Iob, Lacey, and Steptoe (2020), who found that individual types of abuse may have different effects on inflammation; however, if studies are limited in their ability to interrogate specific abuse subtypes or sex differences, then the association may be missed.

In turning to depressive symptoms, our models confirm prior research that has broadly identified experiences of emotional abuse as being highly relevant for depression (Christ et al., 2019; O’Shields, Graves, & Mowbray, 2023), and this finding was replicated in all of our models. However, we found several notable differences among other predictors – namely, that other forms of childhood maltreatment were significantly associated with depressive symptoms but only for women, and that exposure to suicide was associated with depressive symptoms but only for men. Critically, our analyses accounted for early-life depressive symptoms, and therefore, the effects of ACEs can be considered as accounting for increases in depression over time. In this context, emotional abuse, physical abuse, sexual abuse, and emotional neglect were associated with increases in depressive symptoms for women over time.

Interestingly, Desch et al. (2023) identified that latent class analysis-defined clusters of ACEs may have different growth trajectories over time, also using the Add Health dataset. The group with the greatest depressive symptom scores at Wave 1 of the Add Health study exhibited a decrease in depressive symptoms over time, whereas those with a moderate degree of depressive symptoms at Wave 1 showed the greatest increase in depressive symptoms by Wave 4. Critically, the group with declining depressive symptoms had twice the frequency of physical neglect. Our results differ from Desch et al. (2023) in that experiences of physical neglect were not significant predictors in our models, and significance testing for the Physical Neglect × Sex interaction did not survive correction for multiple comparisons. However, it should be noted that results from the present study identified several significant covariance associations with emotional abuse, physical abuse, and emotional neglect, each of which was associated with greater depressive symptoms at Wave 4. Further analysis of ACE × ACE interactions may yield insights into different developmental trajectories, particularly across ACEs characterized by abuse versus neglect.

### A stressor characteristics approach to ACEs

Another notable contribution of this study is that it helps bridge the gap between studies that collapse ACEs into a single index and those that use a dimensionality approach. Indeed, ACEs have been found to be positively associated with depressive symptoms in adulthood (Chapman et al., 2004; Desch et al., 2023; Merrick et al., 2017); however, an alternative approach considers that different ACEs can be differently characterized along the dimensions of deprivation and threat (McLaughlin & Sheridan, 2016). Whereas ACEs strongly characterized by threat (e.g., community violence, abuse) may be more likely to activate neural and biological threat-response pathways (O’Donovan, Slavich, Epel, & Neylan, 2013), those characterized by deprivation may be more likely to affect aspects of neurodevelopment related to associative learning and aspects of executive functioning (McLaughlin & Sheridan, 2016; Sheridan, Peverill, Finn, & McLaughlin, 2017; McLaughlin, Sheridan, & Nelson, 2017). Deficits in executive functioning have been related to greater rumination in depression and greater social stress-induced inflammatory reactivity in healthy individuals (Quinn, Stanton, Slavich, & Joormann, 2020; Snyder, 2012); therefore, when an individual who experiences neglect also experiences threats such as abuse, the neurocognitive and biological threat response may be prolonged.

### Implications for intervention and prevention

Understanding the differing effects of ACEs can also have important implications for intervention and prevention efforts. As noted above, ACEs characterized by deprivation may have lasting effects on neurodevelopment that may affect the experience of threats, and an early referral to appropriate services is thus critical. A meta-analysis of 40 studies evaluating the effectiveness of child physical abuse and neglect prevention programs in families with children ages 0–3 years old found they were effective at reducing the risk factors related to abuse as well as total reports of abuse (Geeraert, Van den Noortgate, Grietens, & Onghena, 2004). However, few providers screen for ACEs or make ACE-based referrals. This is unfortunate, as ACE screening can be conducted in primary care settings in 1–2 minutes, and engaging caregivers in ACE screening has been found to be related to fewer instances of psychological aggression and physical violence (McBain et al., 2023). Moreover, parents have reported positive experiences around discussing ACEs with primary care providers; therefore, discussing how ACEs affect long-term health would most likely be well received by parents (McBain et al., 2023).

If the covariance associations elucidated in the present data replicate, one possibility is that if parents eliminate or reduce substance use in the household, they may be able to reduce the odds of their children experiencing sexual abuse, which is associated with higher CRP levels, particularly among males (Iob et al., 2022). Furthermore, early screening for ACEs could help identify high-risk household environments and potentially help prevent ACEs from occurring during and following the pubertal transition, during which time females are increasingly vulnerable to inflammation due to the effects of both endogenous and exogenous sex hormones (Mengelkoch & Slavich, 2024; Slavich & Sacher, 2019). Given that females are twice as likely to develop depression during this time, routine ACE screening of adolescents could also help direct youth toward treatments that have been found to have both antidepressant and anti-inflammatory effects, such as SSRIs, mindfulness meditation, or cognitive behavior therapy (Black & Slavich, 2016; Galecki, Mossakowska-Wójick, & Talarowska, 2018; Shields, Spahr, & Slavich, 2020; Slavich & Sacher, 2019). More broadly, effective early reduction of inflammation and depressive symptoms may be able to reduce the effects of ACEs on stress and immune processes, thus decreasing the risk for depression and other inflammation-related health problems as individuals age (Kim et al., 2024; Slavich, Mondelli, & Moriarity, in press).

An additional important finding from the present data is the strong association observed between exposure to suicide and greater depressive symptoms among males. Although the model with the full sample revealed an association between exposure to suicide and depressive symptoms, the association was not significant in the female-only model. Conversely, the association between exposure to suicide and depressive symptoms was significant in the male-only model, and the standardized beta was nearly four times greater than in the model based on the total sample. One potential explanation for suicide exposure being associated with greater depressive symptoms in males, but not females, is the context in which suicide exposure occurs. Exploring covariances among ACEs in the male-only model indicated significant covariance between suicide exposure and emotional neglect, suicide exposure and physical abuse, and suicide exposure and emotional abuse. However, the only significant covariance with suicide exposure in the female-only model was with emotional abuse, which was weaker in magnitude relative to the male-only model. Thus, for males, suicide exposure was more likely to occur in scenarios in which social bonds within the family may be weaker. As others have demonstrated, when adolescents experience the death of a friend by suicide, they too are at an increased risk for suicide as well as depressive symptoms (Bearman & Moody, 2004; Gijzen et al., 2021). Social support may help buffer against the risk for suicide and the associated depressive symptoms, and therefore females may be less likely to experience depressive symptoms in relation to suicide exposure, as they were less likely to occur in the context of emotional neglect, emotional abuse, or physical abuse (Kleiman, Riskind, & Schaefer, 2014).

Notably, our decision to keep ACEs as individual adversities enables us to compare our findings to other studies that have focused on a single form of adversity. For example, another recent article from the Add Health study found that parental incarceration portends lasting increases in CRP in children (Tung et al., 2023). However, Tung et al. (2023) did not control for other ACEs aside from including a single measure of childhood maltreatment. In contrast, we adopted an approach to indexing ACEs that is already implemented in the Add Health dataset, in addition to path modeling to control for associations between ACEs. Using this method, we were able to confirm that parental incarceration is associated with increased CRP in children, even while controlling for a broader scope of ACEs. At the same time, our data show that parental incarceration also had the strongest covariance association with in-home substance use. Therefore, although our results are consistent with the main finding of Tung et al. (2023) that parental incarceration has lasting effects on inflammation, our data point toward a need for reducing carceral approaches to dealing with substance use problems. At present, the U.S. has the highest incarceration rate in the world, and around 1 in 5 of those who are arrested have a drug-related offense (Ohringer, Ezer, & Serota, 2020; Sawyer & Wagner, 2024). Policy-level changes such as the decriminalization of cannabis have resulted in lower drug-related charges without any increase in the prevalence of cannabis use, and the wide adoption of such policies could lead to improved health outcomes for inflammation-related diseases in the U.S. (Grucza et al., 2018).

### Inflammation and depression

Interestingly, we did not find associations between CRP and depressive symptoms. Theoretical reasoning seems to support that an increase in CRP should be associated with depressive symptoms. However, Iob et al. (2022) also did not find relations between CRP and depressive symptoms in their cohort study of adolescents transitioning into young adulthood. These investigators argued that a potential reason for this lack of association may be due to the cohort being too young for the association to emerge. However, the present study was still unable to identify such an association despite following participants into their 30s. One reason for this lack of an expected association may be that we did not account for the effects of recent life stressor exposure at the time that CRP and depressive symptoms were assessed. Evidence from Metcalf et al. (2024) identified that women who experienced greater recent stress and had a history of experiencing more ACEs had a significant association between CRP and depressive symptoms. Alternatively, individuals with a current MDE in the context of a history of childhood maltreatment, as opposed to depressive symptoms, have been found to have higher CRP levels than those without a current MDE (Danese et al., 2009). Therefore, CRP may be more strongly related to depressive symptoms in the context of major depression, whereas other inflammatory molecules, such as IL-6, may be related to depressive symptoms regardless of whether someone is presently experiencing depression. Lastly, the use of antidepressants and anti-inflammatory medications in the context of ACEs may have complex effects, and nearly 40% of our sample reported recently using some form of anti-inflammatory medication. This medication use may have, in turn, made it difficult to detect an association between ACEs and CRP or between CRP and depressive symptoms.

### Strengths and limitations

The present study has several strengths. First, the data are from a nationally representative sample of the United States population. Prior studies have used data from an English sample or a sample of United States college students (Iob et al., 2022; Kim et al., 2019), limiting the generalizability of the results. Second, including individual ACEs – rather than collapsing them into an index – provides new insights into the complexities of the relation between stressor characteristics and health. Namely, our analyses highlight the direct effect of specific ACEs on CRP and depressive symptoms and also demonstrate how ACEs cluster together. Therefore, the results may point toward novel intervention and prevention strategies that would have been missed if we had studied the effects of a single ACE in isolation or used clustering-based analytic strategies, such as latent class analysis (Desch et al., 2023; Tung et al., 2023). Third, our analyses investigated associations between ACEs, CRP, and depressive symptoms in the total sample as well as in sex-disaggregated models. This analytic approach highlighted not only the potential sex differences in how ACEs, inflammatory activity, and depression are interrelated, but also what other models may be missing when analyses are conducted only on an aggregate sample of males and females.

Several limitations should also be noted. First, although our analytic strategy focused on identifying inflammatory mechanisms linking ACEs and depressive symptoms, repeated measures approaches are needed to extend the present results and investigate how ACEs alter longitudinal bi-directional associations between inflammation and depressive symptoms over time. Second, although CRP is commonly used to index overall inflammation, has been associated with depression, and is related to other inflammatory markers including IL-6 (Furman et al., 2019), it is still a single inflammatory marker. Future research should thus extend this work to include other inflammatory markers, including IL-6 and TNF-α. Furthermore, because CRP is often used as a measure of chronic, basal inflammation, future studies may benefit from exploring how different ACEs affect stress responsivity as measured by proinflammatory cytokines using laboratory-based acute stress paradigms, such as the Trier Social Stress Test. Third, although the present study used many control variables and longitudinal data from a representative cohort study of the U.S. population, directionality and causality cannot be inferred. Although participant safety may make experimental designs difficult or impossible to implement in the case of ACEs and their effects on health, future studies may be able to better improve on causal inference through a matched pairs design, comparing depressed and non-depressed or ACE- and non-ACE-exposed individuals.

Fourth, although the CES-D has been used in several studies testing associations between inflammation and depressive symptoms, the 10-item version used in the present study may omit symptoms most likely to be associated with inflammation. In particular, future studies may benefit from using measures of depressive symptoms that better assess sleep disturbance, appetite disturbance, and decreased motivation. Lastly, it should be considered that some measures used in the present study, primarily those assessing aspects of childhood maltreatment, are retrospective. Although ACEs cannot be assessed prospectively, per say, some evidence suggests that retrospective measures of ACEs may overestimate associations with subjective measures of health such as depressive symptoms, further highlighting the need for additional research with strong measures of early adversity (Reuben et al., 2016). Measures such as the Childhood Trauma Questionnaire or the Stress and Adversity Inventory could be implemented in future studies, allowing for a more careful qualification of the frequency and severity of ACEs experienced (Bernstein et al., 2003; Slavich & Shields, 2018).

## Conclusion

In conclusion, ACEs are a major risk factor for depressive symptoms and many other health problems in adulthood, potentially through elevated inflammatory activity. Although we did not find evidence of an association between inflammation and depressive symptoms, complex differences were identified between individual ACEs, CRP, and depressive symptoms. Additionally, sex-disaggregated models point toward commonalities in the effects of emotional abuse and parental incarceration while highlighting the sex-specific effects of other forms of childhood maltreatment and exposure to suicide. Furthermore, ACEs were differentially interrelated for males and females, indicating potential differences in how ACEs cluster together across the sexes. Future intervention and prevention efforts should thus account for these complex interrelations between ACEs, as well as their apparent differential effects on CRP and depressive symptoms, when aiming to improve depression-related outcomes.

## Supporting information

O’shields et al. supplementary material O’shields et al. supplementary material

## Supplementary material

The supplementary material for this article can be found at http://doi.org/10.1017/S0033291725001102.
