# Supplementary material for: Adverse childhood experiences, inflammation, and depression: evidence of sex- and stressor specific effects in a nationally representative longitudinal sample of U.S. adolescents
Source: Psychol Med. 2025 May 13;55:e140. doi: 10.1017/S0033291725001102 (PMC12094661; doi:10.1017/S0033291725001102)
Supplement: O’shields et al. supplementary material [file S0033291725001102sup001.docx]

**Supplementary Materials**

**for**

**Adverse Childhood Experiences, Inflammation, and Depression: Evidence of Sex- and Stressor Specific Effects in a Nationally Representative Longitudinal Sample of U.S. Adolescents**

*Control variables* included several factors known to affect the association between CRP and depressive symptoms (Horn et al., 2018), namely: body mass index at Wave 4, current tobacco cigarette smoking status at Wave 4, presence of a chronic health condition at Wave 4, antidepressant use at Wave 4, anti-inflammatory use at Wave 4, and exogenous sex hormone use at Wave 4 (Horn et al., 2018). Wave 4 BMI was included as a continuous variable, calculated through its typical formulation of kilograms divided by meters squared. Tobacco cigarette smoking status at Wave 4 was included categorically as current smoking status (1) or current non-smoking status (0). Presence of a chronic health condition was included categorically as the presence (1) or absence (0) of heart disease, high blood cholesterol, high triglycerides, high lipids, or evidence of current diabetes (HbA1c > 6.5.%, fasting glucose > 200mg/dl, or antidiabetic medication use). Antidepressant use (SSRI, tricyclics, phenylpiperazine, tetracyclic, SSNRI, or miscellaneous antidepressant) at Wave 4 was included categorically as any antidepressant use (1) or no use (0). Anti-inflammatory use (NSAID and salicylates, cox-2 inhibitors, inhaled corticosteroid, corticotropin/glucocorticoid, antirheumatic/antipsoriatic, or immunosuppressives) at Wave 4 was included categorically as any anti-inflammatory use (1) or no use (0). Exogenous sex hormone use (contraceptives, androgens and anabolic steroids, estrogens, gonadotropins, progestins, sex hormone combinations, or miscellaneous sex hormones) at Wave 4 was included categorically as any sex hormone use (1) or no use (0).

*Demographic variables* included age at Wave 1, racial identity, ethnic identity, and four measures of socioeconomic status: Social Origins Score at Wave 1, Occupational Prestige score at Wave 4, and Neighborhood Socioeconomic Disadvantage score at Waves 1 and 4. Age at Wave 1 was included as a continuous variable with a potential range of 12-21 years. Racial identity was included categorically as White (0), Black (1), Asian and/or Pacific Islander (1), Native American (1), other racial identity (1), or multi-racial identity (1). Ethnic identity was included categorically as Hispanic (1), or non-Hispanic (0). We did not combine racial and ethnic identity so as to better reflect how the data was captured, consistent with U.S. Census, as well as to guidance against the collapse of racial and ethnic data when unnecessary (Hirschman et al., 2000; Ross et al., 2020)

Among socioeconomic variables, social Origins scores were pre-calculated by the Add Health team, using principal components analysis to create a composite measure of parental education, parental occupation, household income, and household receipt of public assistance. Social Origins scores were z-transformed by the Add Health team and included in the present study as a continuous measure (Belsky et al., 2018). Occupational Prestige precalculated by the Add Health team via an average of the Hauser and Warren Occupational Income and Occupational Education Scales. This created a socioeconomic index, with greater scores indicating greater socioeconomic status (Belsky et al., 2018). Neighborhood Socioeconomic Disadvantage at Waves 1 and 4 were based on the proportion of five items: female-headed households, individuals living below the poverty threshold, individuals receiving public assistance, adults with less than a high school education, and adults who were unemployed within the participant’s census tract. Each of these items were then scored on a scale of 1-10 and summed, with a potential range of 5-50 (Belsky et al., 2019).

| Supplementary Figure 1: Wave-by-Wave Breakdown of Variable Collection. The figure presents a wave-by-wave breakdown of when each variable in the present study was collected. Participants who had the opportunity to provide data for each of the variables were included in the study. Neighborhood Socioeconomic Disadvantage was shortened to Neighborhood SES for clearer presentation.  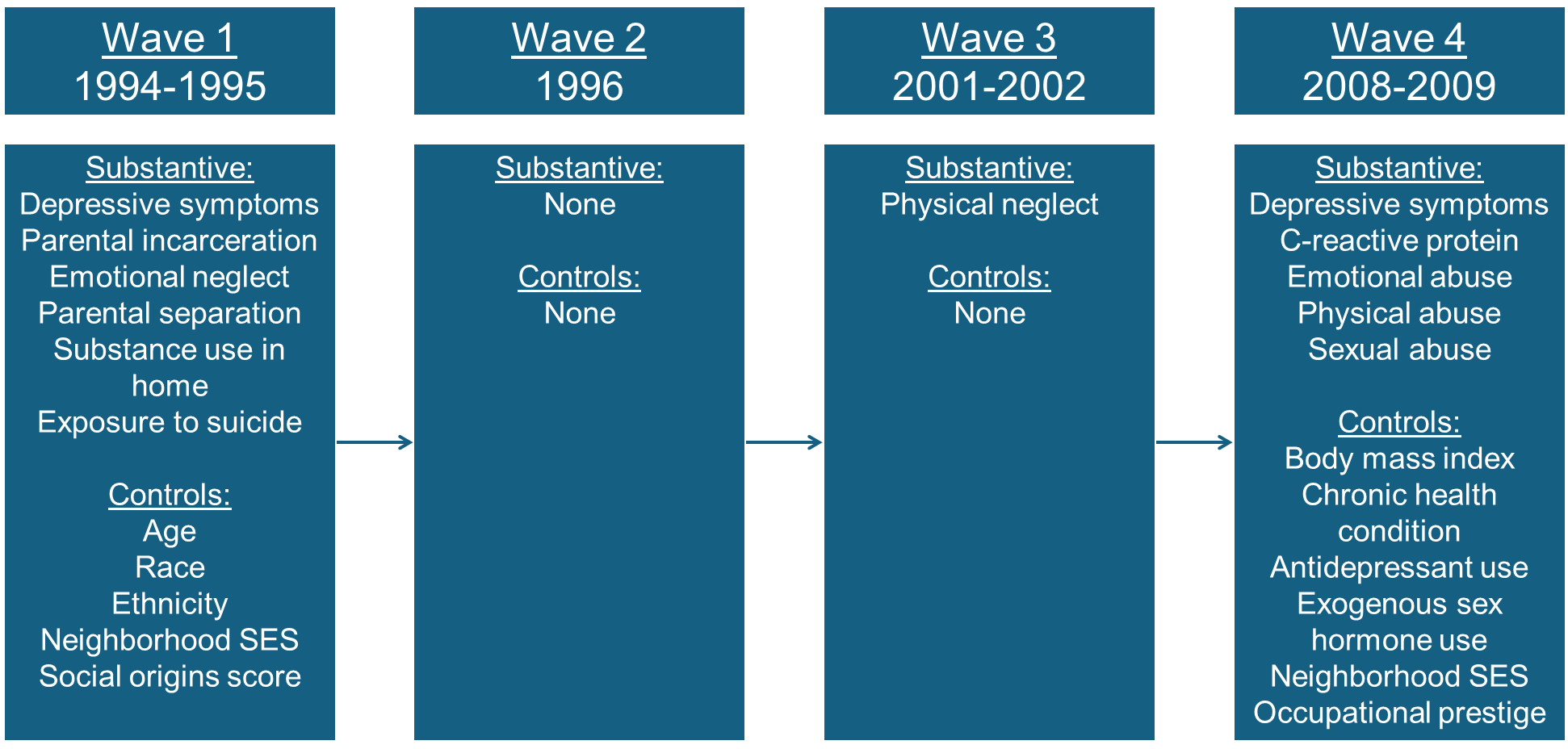 |
| --- |
|  |
|  |

| Supplementary Table 1: ACEs Coding Scheme | | | | |
| --- | --- | --- | --- | --- |
| ACE | Prompt | Coding Scheme | Wave | Source |
| Emotional abuse | “Before your 18^th^ birthday, how often did a parent or other adult caregiver say things that really hurt your feelings or made you feel like you were not wanted or loved?” | Any instance of emotional abuse=1  No history of emotional abuse=0 | 4 | Participant |
| Physical abuse | “Before your 18^th^ birthday, how often did a parent or adult caregiver hit you with a fist, kick you, or throw you down on the floor, into a wall, or down stairs?” | Any instance of physical abuse=1  No history of physical abuse=0 | 4 | Participant |
| Sexual abuse | “Before your 18^th^ birthday how often had one of your parents or other adult care-givers touched you in a sexual way, forced you to touch him or her in a sexual way, or forced you to have sexual relations?” | Any instance of sexual abuse=1  No history of sexual abuse=0 | 4 | Participant |
| Emotional neglect | “Most of the time, your [mother/father] is warm and loving toward you”  “Overall, you are satisfied with your relationship with your [mother/father]”  “You are satisfied with the way your [mother/father] and you communicate with each other.” | Likert scale response for each item ranged from 1 (strongly agree)-5 (strongly disagree). Responses were averaged for mother and father and then summed.  Scores of greater than or equal to 8=1  Scores lower than 8=0 | 1 | Participant |
| Physical neglect | “How often had your parents or other adult care-givers not taken care of your basic needs, such as keeping you clean or providing food or clothing?” | Any instance of physical neglect =1  No history of physical neglect=0 | 3 | Participant |
| Parental incarceration | “(Has/did) your [biological mother/  biological father/mother figure/father figure] ever  (spent/spend) time in jail or prison?” | Any time in jail or prison=1  No time in jail or prison=0 | Parent | Participant |
| Parental separation | “What is your current marital status” | Divorced or separated=1  Single (never married), married, widowed=0 | Parent | Parent |
| Substance use in the home | “Does respondent’s biological mother currently have the following health problem: Alcoholism”  “Does respondent’s biological father currently have the following health problem: Alcoholism”  “Are illegal drugs easily available to you in your home” | Any parental report of alcoholism or self-report access to drugs in the home=1  No parental report of alcoholism or self-report access to drugs in the home=0 | Parent/1 | Parent |
| Community violence | “During the past 12 months, how often did you see someone shoot or stab another person?”  “During the past 12 months, how often did someone pull a knife or gun on you?”  “During the past 12 months, how often did someone shoot or stab you?”  “During the past 12 months, how often did someone cut or stab you?” | Any history of community violence in the past 12 months=1  No history of community violence in the past 12 months=0 | 1 | Participant |
| Exposure to suicide | “Have any of your friends tried to kill themselves during the past 12 months?”  “Have any of them succeeded?” | Attempt and death by suicide in the past 12 months=1  No death by suicide in the past 12 months=0 | 1 | Participant |

| Supplementary Table 2: Depressive Symptoms as Measured by the CES-D and Availability of Each Question by Add Health Wave | | | |
| --- | --- | --- | --- |
| Item | Prompt | Wave | |
|  |  | 1 | 4 |
| 1 | You were bothered by things that usually don’t bother you | X | X |
| 2 | You did not feel like eating; your appetite was poor | X |  |
| 3 | You felt that you could not shake off the blues even with help from your family or friends | X | X |
| 4 | You felt you were just as good as other people | X | X |
| 5 | You had trouble keeping your mind on what you were doing | X | X |
| 6 | You felt depressed | X | X |
| 7 | You felt that you were too tired to do things | X | X |
| 8 | You felt hopeful about the future | X |  |
| 9 | You thought your life had been a failure | X |  |
| 10 | You felt fearful | X |  |
| 11 | My sleep was restless |  |  |
| 12 | You were happy | X | X |
| 13 | You talked less than usual | X |  |
| 14 | You felt lonely | X |  |
| 15 | People were unfriendly | X |  |
| 16 | You enjoyed life | X | X |
| 17 | You had crying spells |  |  |
| 18 | You felt sad | X | X |
| 19 | You felt that people dislike you | X | X |
| 20 | It was hard to get started doing things | X |  |
| 21 | You felt life was not worth living | X |  |
| Items 11 and 17 are typically included in the 20-item version of the CES-D but were not implemented in the Add Health version.  Item 21 is not typically included in the 20-item version of the CES-D but was included for Add Health  Waves 1 & 2 are 19 items, Wave 3 is 10 items, Wave 4 is 9 items, and Wave 5 is 5 items | | | |

| Table 3: Standardized Coefficients for non-Recursive Path Model of Different ACEs Predicting Depressive Symptoms and CRP Across the Total Sample, Females Only, and Males Only | | | | | | |
| --- | --- | --- | --- | --- | --- | --- |
|  | Total | | Females | | Males | |
|  | Depressive Symptoms | CRP | Depressive Symptoms | CRP | Depressive Symptoms | CRP |
|  | β | β | β | β | Β | β |
| ACE |  | |  |  |  |  |
| Emotional abuse | 0.08*** | -0.05*** | 0.07*** | -0.06** | 0.09** | -0.08** |
| Physical abuse | 0.02 | -0.01 | 0.05** | -0.01 | -0.05 | -0.01 |
| Sexual abuse | 0.05** | -0.01 | 0.06** | -0.01 | 0.01 | 0.12*** |
| Physical neglect | -0.03 | -0.01 | -0.04 | -0.03 | -0.04 | 0.05 |
| Emotional neglect | 0.03 | -0.01 | 0.05** | 0.01 | -0.01 | -0.04 |
| Parental separation | 0.01 | -0.02 | 0.01 | -0.02 | -0.01 | 0.01 |
| In-home substance use | 0.01 | 0.02 | 0.01 | 0.03 | 0.01 | 0.04 |
| Parental incarceration | -0.01 | 0.08*** | -0.02 | 0.08*** | 0.04 | 0.7* |
| Suicide exposure | 0.05** | 0.01 | -0.01 | 0.01 | 0.19*** | -0.01 |
| Community violence | -0.01 | -0.01 | -0.01 | -0.01 | -0.01 | -0.01 |
| ACE: adverse childhood experience; CRP: C-reactive protein  **p* < 0.05, ***p* < 0.01, ****p* < 0.001 | | | | | | |

| Supplementary Table 4: Covariance Between ACEs and Between the Residuals for Depressive Symptoms and C-Reactive Protein for the Total Sample Multivariate Model | | | | | | | | | | | | | | | | | |
| --- | --- | --- | --- | --- | --- | --- | --- | --- | --- | --- | --- | --- | --- | --- | --- | --- | --- |
|  |  | 1 | 2 | 3 | 4 | 5 | 6 | 7 | 8 | 9 | 10 | 11 | 12 | 13 | 14 | 15 | 16 |
| Depression | 1 | - |  |  |  |  |  |  |  |  |  |  |  |  |  |  |  |
| C-reactive protein | 2 | 0.03 | - |  |  |  |  |  |  |  |  |  |  |  |  |  |  |
| Emotional abuse | 3 | - | - | - |  |  |  |  |  |  |  |  |  |  |  |  |  |
| Physical abuse | 4 | - | - | **0.35** | - |  |  |  |  |  |  |  |  |  |  |  |  |
| Sexual abuse | 5 | - | - | **0.14** | **0.16** | - |  |  |  |  |  |  |  |  |  |  |  |
| Physical neglect | 6 | - | - | **0.05** | **0.10** | 0.02 | - |  |  |  |  |  |  |  |  |  |  |
| Emotional neglect | 7 | - | - | **0.20** | **0.14** | **0.09** | 0.03 | - |  |  |  |  |  |  |  |  |  |
| Parental separation | 8 | - | - | **0.09** | **0.08** | **0.05** | **0.06** | **0.04** | - |  |  |  |  |  |  |  |  |
| In-home substance use | 9 | - | - | **0.12** | **0.07** | **0.08** | **0.07** | **0.07** | **0.20** | - |  |  |  |  |  |  |  |
| Parental incarceration | 10 | - | - | **0.11** | **0.10** | **0.08** | **0.14** | **0.14** | **0.14** | **0.24** | - |  |  |  |  |  |  |
| Suicide exposure | 11 | - | - | **0.06** | **0.05** | 0.01 | -0.03 | **0.05** | 0.01 | 0.01 | -0.01 | - |  |  |  |  |  |
| Community violence | 12 | - | - | **0.06** | **0.09** | 0.01 | **0.09** | **0.06** | **0.13** | **0.08** | 0.02 | **0.14** | - |  |  |  |  |
| Family SES | 13 | - | - | **-0.08** | **-0.12** | **-0.08** | **-0.08** | **-0.05** | **-0.23** | **-0.12** | **-0.23** | -0.01 | **-0.13** | - |  |  |  |
| Neighborhood | 14 | - | - | 0.01 | 0.02 | **0.06** | **0.05** | 0.03 | **0.08** | 0.03 | **0.11** | 0.01 | **0.11** | **-0.37** | - |  |  |
| Occupational Prestige | 15 | - | - | - | - | - | - | - | - | - | - | - | - | **0.20** | - | - |  |
| Neighborhood | 16 | - | - | - | - | - | - | - | - | - | - | - | - | - | **0.28** | **-0.14** | - |
| Standardized coefficients for covariances in the multivariate model  Associations between items 3-16 are covariances  Association between items 1 and 2 is the covariance between residuals  Bolded coefficients are significant at least at the *p* < 0.05 level SES = Socioeconomic Status | | | | | | | | | | | | | | | | | |

| Supplementary Table 5: Covariance Between ACEs and Between the Residuals for Depressive Symptoms and C-Reactive Protein for the Male Sample Multivariate Model | | | | | | | | | | | | | | | | | | |
| --- | --- | --- | --- | --- | --- | --- | --- | --- | --- | --- | --- | --- | --- | --- | --- | --- | --- | --- |
|  |  | 1 | 2 | 3 | 4 | 5 | 6 | 7 | 8 | 9 | 10 | 11 | 12 | 13 | 14 | 15 | 16 |  |
| Depression | 1 | - |  |  |  |  |  |  |  |  |  |  |  |  |  |  |  |  |
| C-reactive protein | 2 | 0.05 | - |  |  |  |  |  |  |  |  |  |  |  |  |  |  |  |
| Emotional abuse | 3 | - | - | - |  |  |  |  |  |  |  |  |  |  |  |  |  |  |
| Physical abuse | 4 | - | - | **0.35** | - |  |  |  |  |  |  |  |  |  |  |  |  |  |
| Sexual abuse | 5 | - | - | **0.16** | **0.18** | - |  |  |  |  |  |  |  |  |  |  |  |  |
| Physical neglect | 6 | - | - | 0.05 | 0.05 | 0.07 | - |  |  |  |  |  |  |  |  |  |  |  |
| Emotional neglect | 7 | - | - | **0.17** | **0.16** | **0.09** | **0.06** | - |  |  |  |  |  |  |  |  |  |  |
| Parental separation | 8 | - | - | **0.06** | 0.01 | -0.02 | **0.10** | 0.02 | - |  |  |  |  |  |  |  |  |  |
| In-home substance use | 9 | - | - | **0.11** | **0.08** | **0.17** | **0.08** | **0.12** | **0.28** | - |  |  |  |  |  |  |  |  |
| Parental incarceration | 10 | - | - | **0.14** | **0.08** | **0.08** | **0.21** | **0.08** | **0.21** | **0.32** | - |  |  |  |  |  |  |  |
| Suicide exposure | 11 | - | - | **0.10** | **0.11** | 0.04 | -0.05 | **0.15** | -0.01 | 0.01 | -0.03 | - |  |  |  |  |  |  |
| Community violence | 12 | - | - | 0.01 | 0.02 | -0.01 | **0.10** | **0.07** | **0.11** | **0.08** | -0.03 | **0.14** | - |  |  |  |  |  |
| Family SES | 13 | - | - | -0.05 | **-0.14** | -0.06 | **-0.07** | -0.04 | **-0.27** | **-0.20** | **-0.23** | 0.01 | **-0.12** | - |  |  |  |  |
| Neighborhood | 14 | - | - | -0.01 | -0.01 | 0.01 | 0.04 | 0.04 | 0.05 | 0.06 | **0.09** | 0.04 | **0.18** | **-0.36** | - |  |  |  |
| Occupational Prestige | 15 | - | - | - | - | - | - | - | - | - | - | - | - | **0.20** | - | - |  |  |
| Neighborhood | 16 | - | - | - | - | - | - | - | - | - | - | - | - | - | **0.27** | **-0.16** | - |  |
| Standardized coefficients for covariances in the multivariate model  Associations between items 3-16 are covariances  Association between items 1 and 2 is the covariance between residuals  Bolded coefficients are significant at least at the *p* < 0.05 level SES = Socioeconomic Status | | | | | | | | | | | | | | | | | | |

| Supplementary Table 6: Covariance Between ACEs and Between the Residuals for Depressive Symptoms and C-Reactive Protein for the Female Sample Multivariate Model | | | | | | | | | | | | | | | | | |
| --- | --- | --- | --- | --- | --- | --- | --- | --- | --- | --- | --- | --- | --- | --- | --- | --- | --- |
|  |  | 1 | 2 | 3 | 4 | 5 | 6 | 7 | 8 | 9 | 10 | 11 | 12 | 13 | 14 | 15 | 16 |
| Depression | 1 | - |  |  |  |  |  |  |  |  |  |  |  |  |  |  |  |
| C-reactive protein | 2 | -0.01 | - |  |  |  |  |  |  |  |  |  |  |  |  |  |  |
| Emotional abuse | 3 | - | - | - |  |  |  |  |  |  |  |  |  |  |  |  |  |
| Physical abuse | 4 | - | - | **0.35** | - |  |  |  |  |  |  |  |  |  |  |  |  |
| Sexual abuse | 5 | - | - | **0.13** | **0.16** | - |  |  |  |  |  |  |  |  |  |  |  |
| Physical neglect | 6 | - | - | **0.08** | **0.13** | 0.02 | - |  |  |  |  |  |  |  |  |  |  |
| Emotional neglect | 7 | - | - | **0.21** | **0.14** | **0.09** | 0.03 | - |  |  |  |  |  |  |  |  |  |
| Parental separation | 8 | - | - | **0.10** | **0.12** | **0.08** | 0.04 | **0.05** | - |  |  |  |  |  |  |  |  |
| In-home substance use | 9 | - | - | **0.13** | **0.06** | **0.06** | **0.07** | **0.08** | **0.16** | - |  |  |  |  |  |  |  |
| Parental incarceration | 10 | - | - | **0.10** | **0.10** | **0.08** | **0.09** | **0.04** | **0.10** | **0.20** | - |  |  |  |  |  |  |
| Suicide exposure | 11 | - | - | **0.04** | 0.01 | -0.01 | -0.01 | 0.01 | 0.02 | 0.02 | 0.01 | - |  |  |  |  |  |
| Community violence | 12 | - | - | **0.11** | **0.14** | 0.02 | **0.05** | **0.07** | **0.15** | **0.08** | **0.05** | **0.14** | - |  |  |  |  |
| Family SES | 13 | - | - | **-0.10** | **-0.11** | **-0.09** | **-0.08** | **-0.05** | **-0.21** | **-0.07** | **-0.23** | -0.01 | **-0.14** | - |  |  |  |
| Neighborhood Wave 1 | 14 | - | - | 0.01 | **0.04** | **0.07** | **0.06** | 0.02 | **0.10** | 0.01 | **0.12** | -0.01 | **0.07** | **-0.37** | - |  |  |
| Occupational Prestige | 15 | - | - | - | - | - | - | - | - | - | - | - | - | **0.21** | - | - |  |
| Neighborhood Wave 4 | 16 | - | - | - | - | - | - | - | - | - | - | - | - | - | **0.30** | **-0.12** | - |
| Standardized coefficients covariances in the multivariate model  Associations between items 3-16 are covariances  Association between items 1 and 2 is the covariance between residuals  Bolded coefficients are significant at least at the *p* < 0.05 level SES = Socioeconomic Status | | | | | | | | | | | | | | | | | |

References

Belsky, D. W., Caspi, A., Arseneault, L., Corcoran, D. L., Domingue, B. W., Harris, K. M., …, & Odgers, C. L. (2019). Genetics and the geography of health, behaviour and attainment. *Nature Human Behaviour*, *3*(6), 576-586. https://doi.org/10.1038/s41562-019-0562-1

Belsky, D. W., Domingue, B. W., Wedow, R., Arseneault, L., Boardman, J. D., Caspi, A., …, & Harris, K. M. (2018). Genetic analysis of social-class mobility in five longitudinal studies. *Proceedings of the National Academy of Sciences*, *115*(31), E7275-E7284. https://doi.org/doi:10.1073/pnas.1801238115

Hirschman, C., Alba, R. & Farley, R. The meaning and measurement of race in the U.S. census: Glimpses into the future. *Demography,* 37, 381–393 (2000). <https://doi.org/10.2307/2648049>

Horn, S. R., Long, M. M., Nelson, B. W., Allen, N. B., Fisher, P. A., & Byrne, M. L. (2018). Replication and reproducibility issues in the relationship between C-reactive protein and depression: A systematic review and focused meta-analysis. *Brain, Behavior, and Immunity*, *73*, 85-114. https://doi.org/10.1016/j.bbi.2018.06.016

Ross, P.T., Hart-Johnson, T., Santen, S.A., Zaidi, N. L. B. (2020)*.* Considerations for using race and ethnicity as quantitative variables in medical education research. *Perspectives on Medical Education,* 9, 318–323. https://doi.org/10.1007/s40037-020-00602-3
